# Supplementary material for: Structural evolution at the oxidative and reductive limits in the first electrochemical cycle of Li1.2Ni0.13Mn0.54Co0.13O2
Source: Nat Commun. 2020 Mar 6;11:1252. doi: 10.1038/s41467-020-14927-4 (PMC7060333; doi:10.1038/s41467-020-14927-4)
Supplement: Supplementary file 1 — Supplementary Information [file 41467_2020_14927_MOESM1_ESM.pdf]

Supplementary Information

Structural evolution at the oxidative and reductive limits in the first electrochemical cycle of $Li_{1.2}Ni_{0.13}Mn_{0.54}Co_{0.13}O_2$

Wei Yin *et al.*

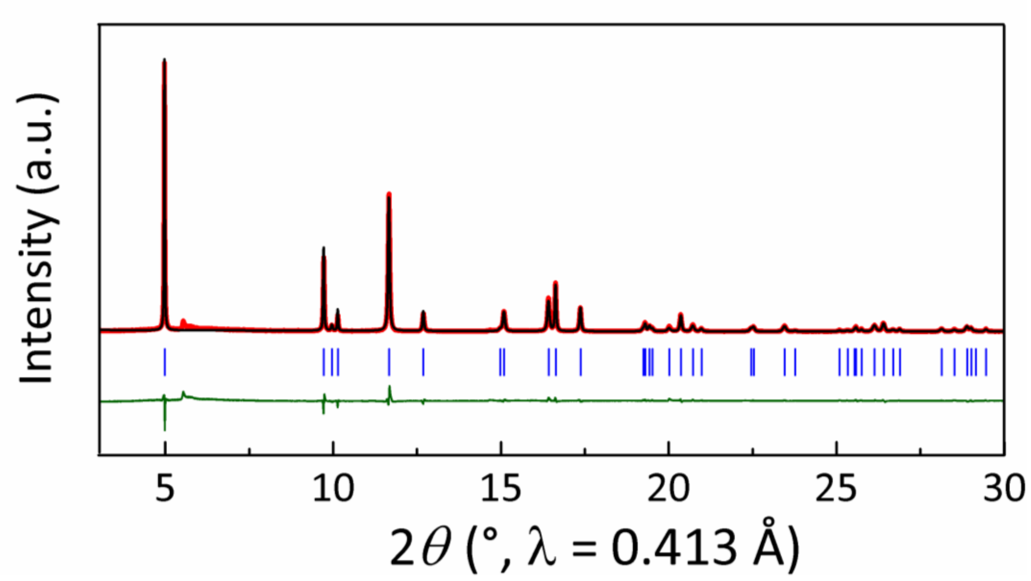

Supplementary Figure 1 Rietveld refinement of the synchrotron XRD pattern of the pristine $Li_{1.2}Ni_{0.13}Mn_{0.54}Co_{0.13}O_2$ phase. The red line, black line and bottom green line represent the observed, calculated XRD patterns and the difference, respectively. Vertical blue tick bars mark the Bragg positions in the $R\bar{3}m$ space group.

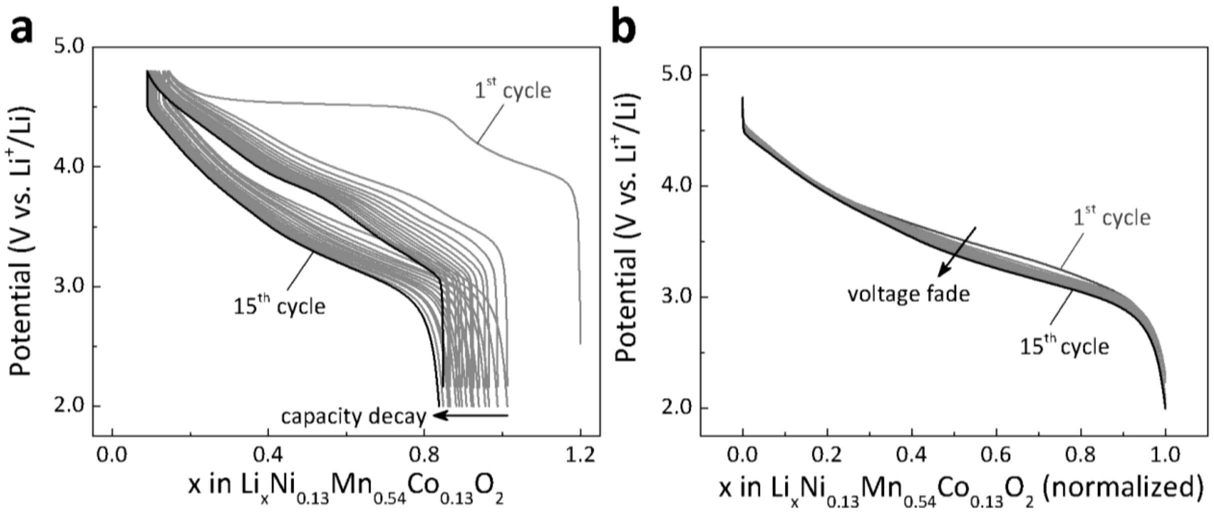

Supplementary Figure 2 Cycling performance of the pristine $Li_{1.2}Ni_{0.13}Mn_{0.54}Co_{0.13}O_2$ phase. a, Potential-composition curves of Li-rich NMC cycled within a potential window of 4.8 - 2.0 V at a current rate of C/5. **b,** Normalized discharge curves of Li-rich NMC cycled within a potential window of 4.8 - 2.0 V at a current rate of C/5, i.e., the Li content (x) were normalized such that the maximum Li content in each discharge is taken as unity (x = 1.01 and 0.84 are taken as unity for the first and fifteenth cycle, respectively.)

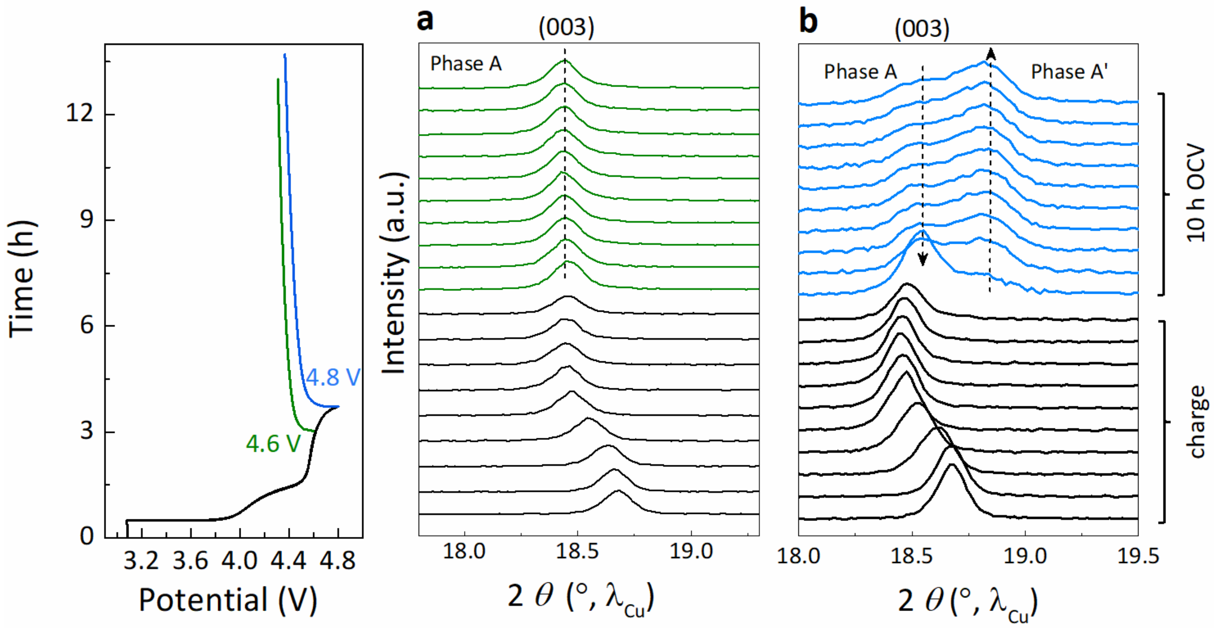

Supplementary Figure 3 Formation of a new phase A' on deep oxidation. *In-situ* XRD study of the first-charge of Li-rich NMC charged to 4.6 V (a) and 4.8 V (b), followed with an open circuit voltage (OCV) step for 10 h. Note that only the (003) Bragg peak is shown here because it represents the major change of the whole XRD pattern. Dash line in panel (**a**) suggests that only the phase A is formed after 4.6 V charge. On the contrary, a new phase A' is grown at the expense of the phase A on further charge to 4.8 V, as indicated by the dashed arrows in panel (**b**). Note that the A → A' phase transition only occurs during the OCV period when intentionally charging the cells at a relatively fast current rate (i.e., C/3), suggesting the slow kinetics for such phase transition.

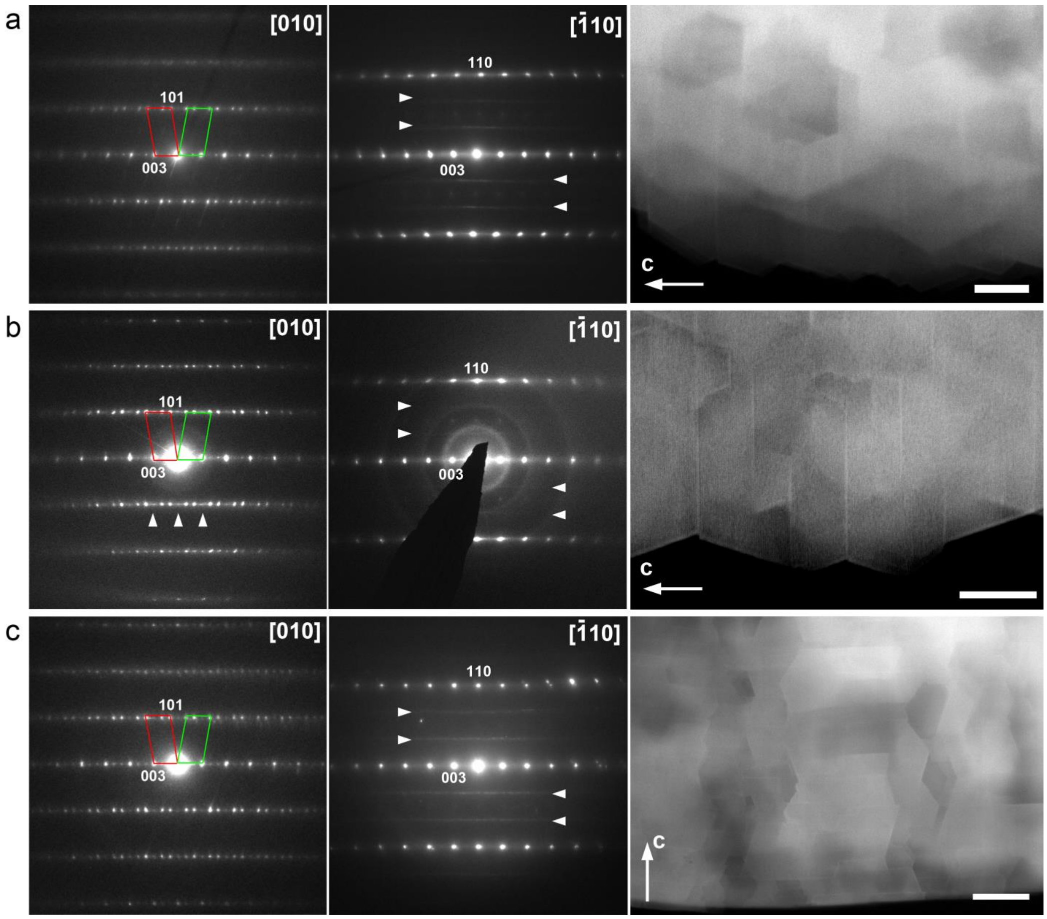

Supplementary Figure 4 Electron diffraction (ED) patterns and low magnification HAADF-STEM images of Li-rich NMC samples: charged to 4.6 V (**a**, the A phase), 4.8 V (**b**, the A' phase) and discharged to 2.0 V (**c**). The ED patterns are indexed with the O3 $R\bar{3}m$ structure. Note the reflection splitting in the *h*0*l*, $h \neq 3n$ reciprocal lattice rows in all [010] ED patterns which is caused by mirror twinning of the O3 $R\bar{3}m$ structure with the (001) twin plane (Supplementary Fig. 6). The primitive reciprocal unit cells of two twinned variants are outlined in red and green in the [010] ED patterns. Twinned domains with few tens of nanometers in thickness along the *c*-axis are clearly visible in low magnification HAADF-STEM images for all three states of charge. Extra spots elongated along the **c*** axis appear between the pairs of bright reflections of the O3 structure in the [010] ED pattern of the A' phase (**b**, marked with vertical arrowheads). These reflections originate from thin domains of the O1 structure, as evidenced with the HAADF-STEM images in Supplementary Fig. 7. [$\bar{1}$10] ED pattern of the A phase (**a**) reveals faint diffuse intensity lines (marked with horizontal arrowheads) from residual short-range "honeycomb" Li-M ordering. These diffuse intensity lines become almost invisible in the [$\bar{1}$10] ED pattern of the A' phase (**b**) indicating significant suppression of the "honeycomb" Li-M ordering, but regain their brightness after discharge to 2.0 V (**c**) due to restoration of this ordering. Weak spots between diffuse intensity lines in the [$\bar{1}$10] ED patterns of Li-rich NMC charged to 4.6 V (**a**) and 4.8 V (**b**) come from the near-surface M cation ordering analyzed in Supplementary Fig. 8. Scale bars in panels **a** and **b** indicate 30 nm, scale bar in panel **c** indicates 50 nm.

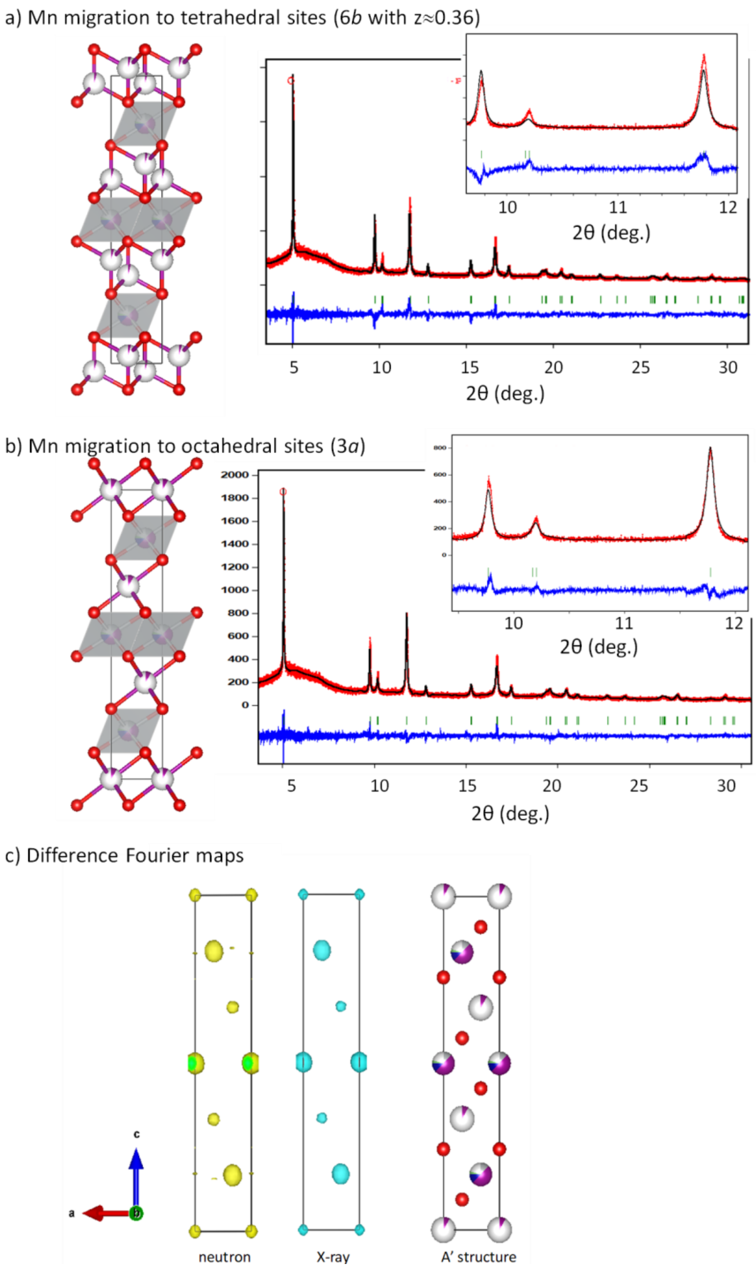

Supplementary Figure 5 Structural determination of phase A'. a) and b) Rietveld refinement using two models: a) with Mn migration in tetrahedral sites (Wyckoff position 6*c* with z close to 0.36), and b) with the same amount of Mn migration to octahedral sites 3*a* (i.e., at the same position as Li in Li layers for the pristine material). The effect on the synchrotron XRD refinement is shown next to the model, with a zoom on peaks (101), (012)/(006) and (104) as inset. The model with Mn in tetrahedral sites presents a refinement which is worsen compared to the one be obtained with Mn in Li octahedral sites. c) : **Difference Fourier Maps generated from neutron and X-ray diffraction patterns of the A' phase,** calculated after having removed all transition metals from the structure (Ni, Mn and Co). On the right-hand side is given a view of the structure as deduced from the combined refinement of the neutron and XRD patterns (oxygen is red, Mn is purple, Li is green, Ni is gray, Co is dark blue).

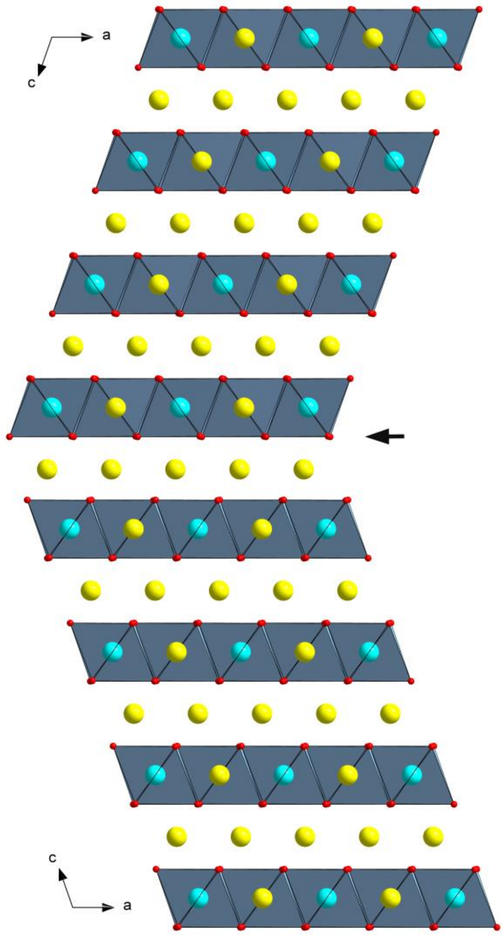

Supplementary Figure 6 **Tentative model of twins in the O3 $R\bar{3}m$ structure**. Two twinned domains viewed along [010] possess mirror-related orientation of the *c*-axis. At the twin plane the "cubic" *ccp* close packing of the O atoms is interrupted by a single "hexagonal" *h* layer (marked with an arrow). The M cations, Li cations and O atoms are shown as cyan, yellow and red spheres, respectively. The MO_6 octahedra are shaded.

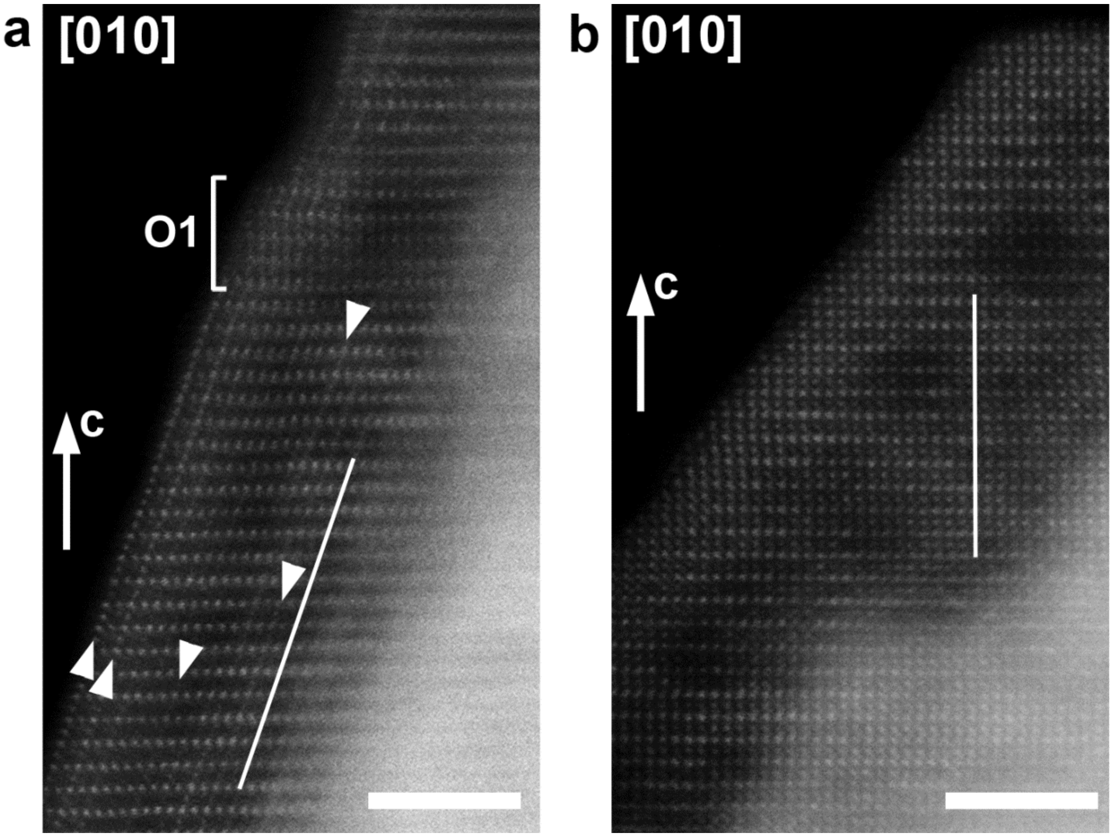

Supplementary Figure 7 Local structure of the A' phase viewed with [010] HAADF-STEM images. a, The near-surface O3 structure: the M layers are horizontally being traced with the rows of bright dots of the M cationic columns. The "cubic" stacking sequence is evidenced with cumulative lateral shift of the $Li_{1-x}M_{2+x}$ layers (marked with a sloped line). The migration of the M cations to the octahedral interlayer sites is visible as a nucleation of the $\{10\bar{2}\}$-oriented TM-cation layer at ~ 60° to the {001}-oriented $Li_{1-x}M_{2+x}$ layers (marked with arrowheads). Thin lamella of the O1 phase is marked with a bracket. **b,** The near-surface structure of an extended O1 domain: the "hexagonal" stacking sequence is evidenced by absence of the lateral displacements of the $Li_{1-x}M_{2+x}$ layers (marked with a vertical line). The migration of the M cations to the interlayer octahedral sites is very pronounced seen by appearance of extra rows of dots between the $Li_{1-x}M_{2+x}$ layers (look brighter). Scale bars indicate 3 nm.

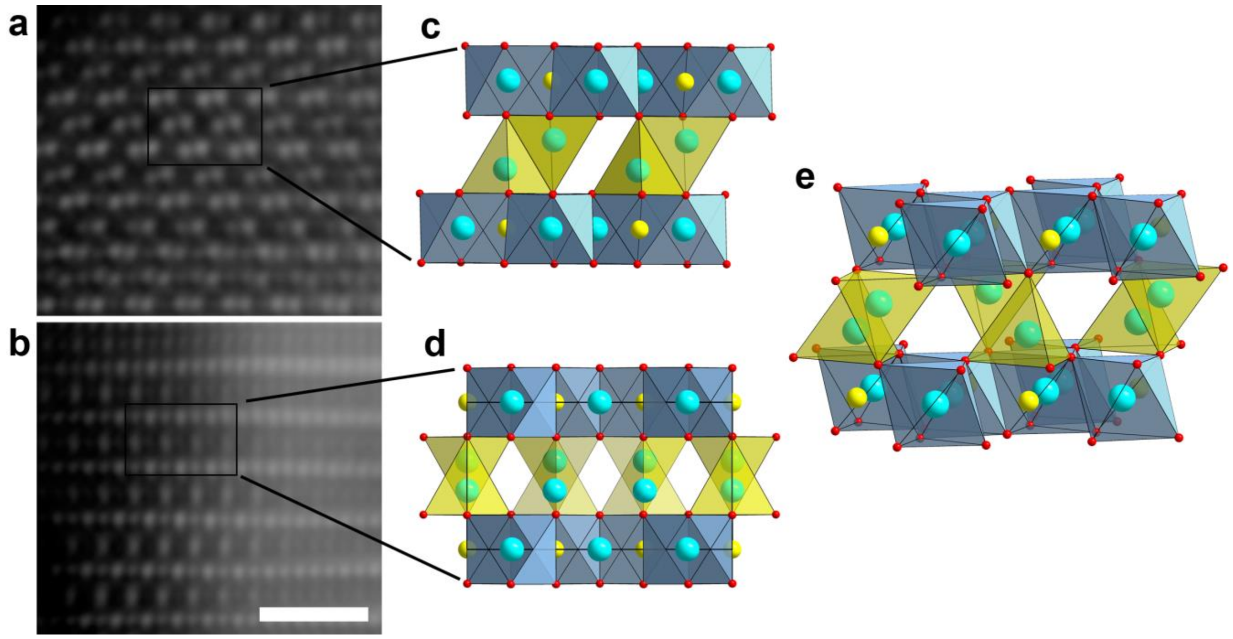

Supplementary Figure 8 Near-surface locally ordered states in the A' phase. a, b, HAADF-STEM images of the ordering of M cations in the tetrahedral interstices between the M layers with pertaining "honeycomb" M cation ordering (the scale bar is of 1 nm). **c, d,** Projected structures corresponding to the cationic positions in the HAADF-STEM images **a** and **b**, respectively. The M cations, Li cations and O atoms are shown as cyan, yellow and red spheres. **e,** The projected structures, in fact, correspond to the same 3D structural arrangements viewed at different directions rotated by 60°. Note that the tetrahedral interstices are linked by common edge and cannot be occupied simultaneously.

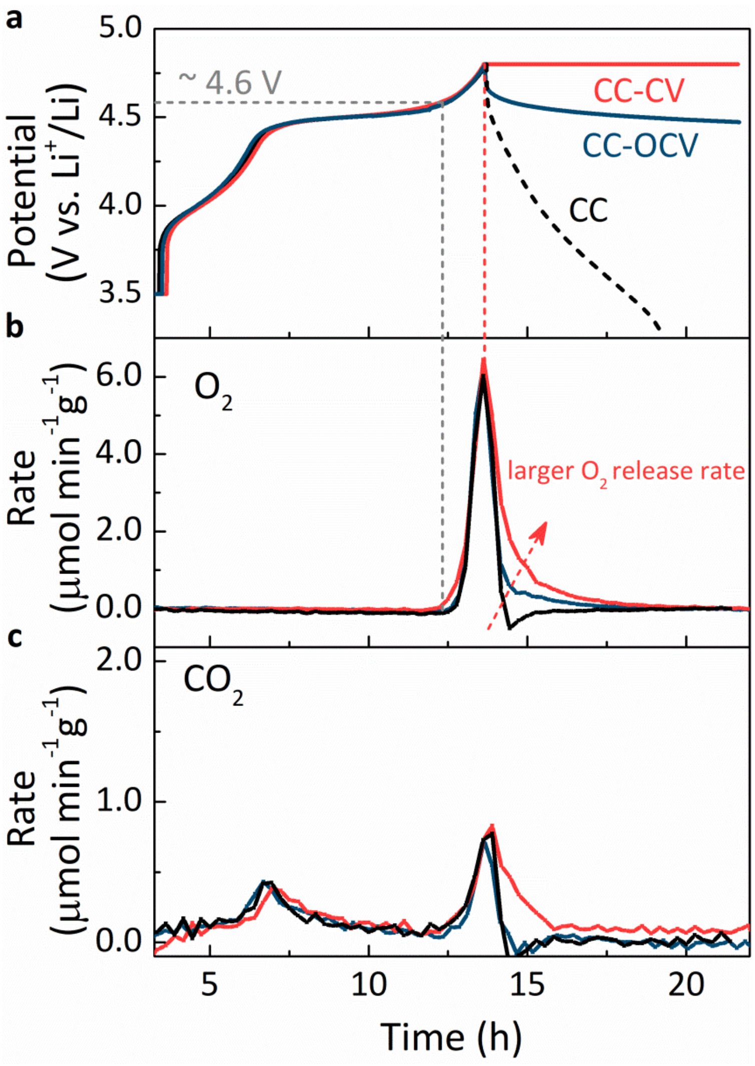

Supplementary Figure 9 Gas release from Li-rich NMC on deep oxidation. OEMS gas analysis during the first constant current (CC) charge of Li-rich NMC followed by a CC discharge (black lines), an OCV step (blue lines) and a constant voltage (CV) hold at 4.8 V (red lines). O_2 (m/z = 32) and CO_2 (m/z = 44) ion currents were recorded and then converted to gas evolution rates. **a,** The potential *vs* time curves (note that the curves are superimposed up to the 4.8 V charge cutoff potential indicating a good reproducibility of the OEMS cells, and the dash black line illustrates partially the subsequent discharge for the sake of comparison) and the evolved rates of O_2 (**b**) and CO_2 (**c**) in the units of μmol $min^{-1}g^{-1}$ with colors quoted the same as in panel (**a**). The grey dash line indicates the onset potential of O_2 evolution. The red line marks the 4.8 V cutoff potential of the CC charge. The red dash arrow reveals that larger O_2 evolution was observed in CC-CV charged cell.

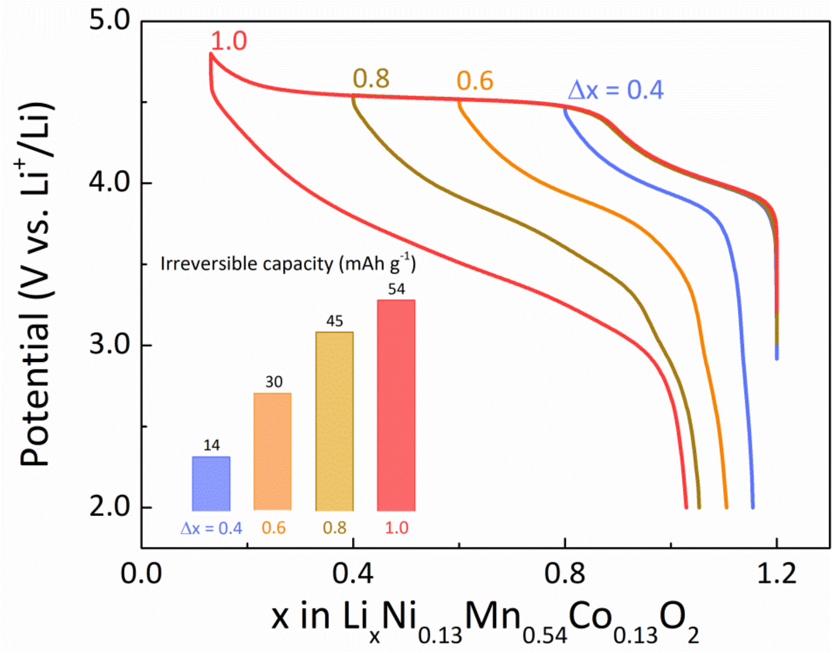

Supplementary Figure 10 Potential-composition profiles of Li-rich NMC obtained by varying the Li^+ removal on charge (Δx) from 0.4 (purple line), 0.6 (orange line), 0.8 (dark yellow line), to 1.0 (red line), while fixing the discharge cutoff potential at 2.0 V. The inset show their corresponding irreversible capacities observed in discharge.

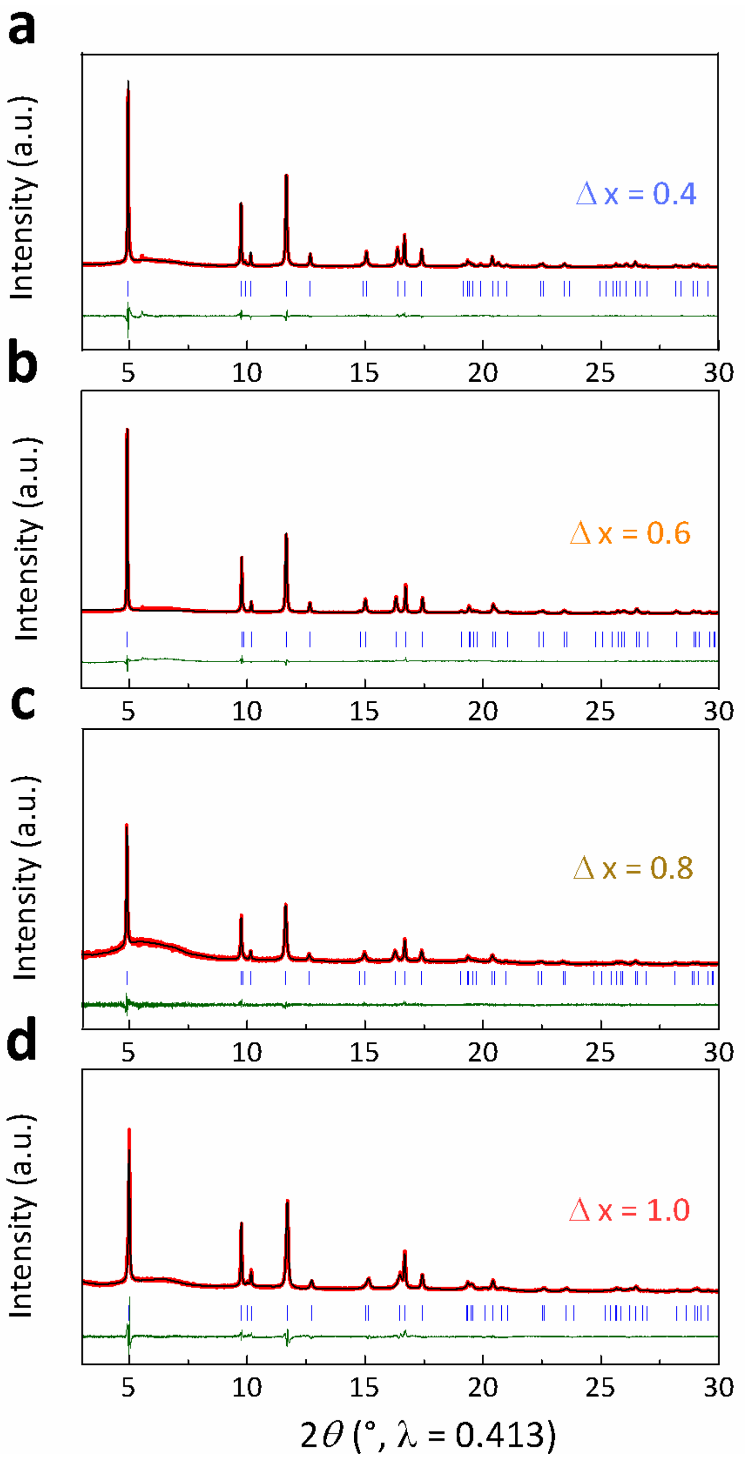

Supplementary Figure 11 Structural evolution of Li-rich NMC in the first charge as a function of state of charge. Rietveld refinements of the synchrotron XRD patterns of Li-rich NMC collected at their charged states with the amount of Li^+ removal (Δx) ranging from 0.4 (**a**), 0.6 (**b**), 0.8 (**c**), and 1.0 (**d**), followed by 120 h OCV. The red line, black line and bottom green line represent the observed, calculated XRD patterns and the difference, respectively. Vertical blue tick bars mark the Bragg positions in the $R\bar{3}m$ space group.

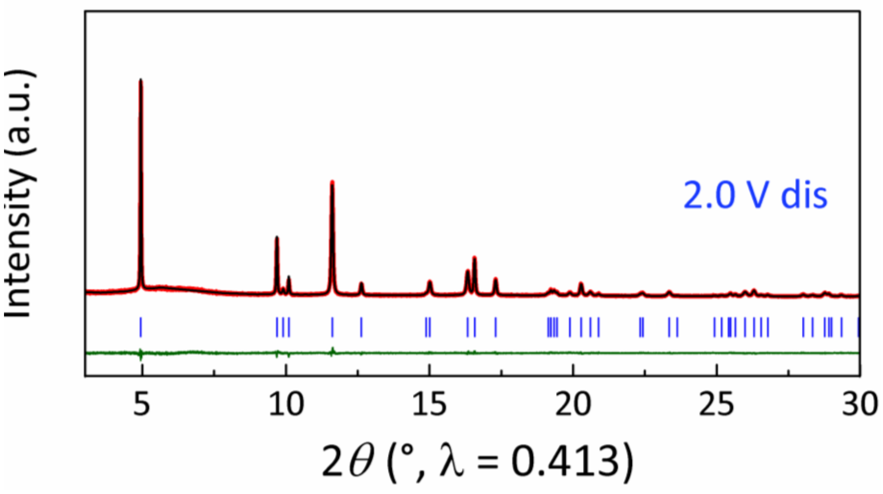

Supplementary Figure 12 Rietveld refinement of the synchrotron XRD pattern of Li-rich NMC charged to 4.8 V and then discharged to 2.0 V. The red line, black line and bottom green line represent the observed, calculated XRD patterns and the difference, respectively. Vertical blue tick bars mark the Bragg positions in the $R\bar{3}m$ space group.

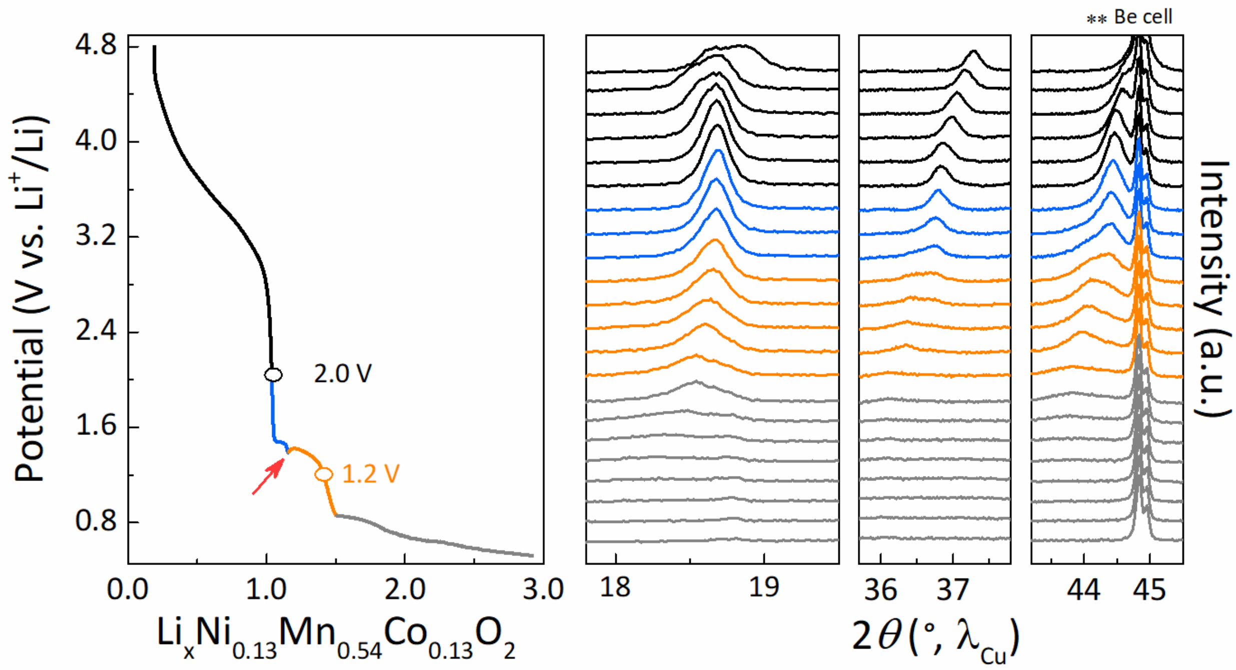

Supplementary Figure 13 Structural evolution of Li-rich NMC on deep discharge from 4.8 to 0.5 V. Galvanostaic discharge curve collected on discharging Li-rich NMC down to 0.5 V after 4.8 V charge (left side) and the corresponding *operando* XRD patterns. Blue and yellow lines show the results before and after the overshooting point (marked by red arrow), respectively. Grey lines reveal that further pushing the discharge down to 0.5 V results in a material amorphization.

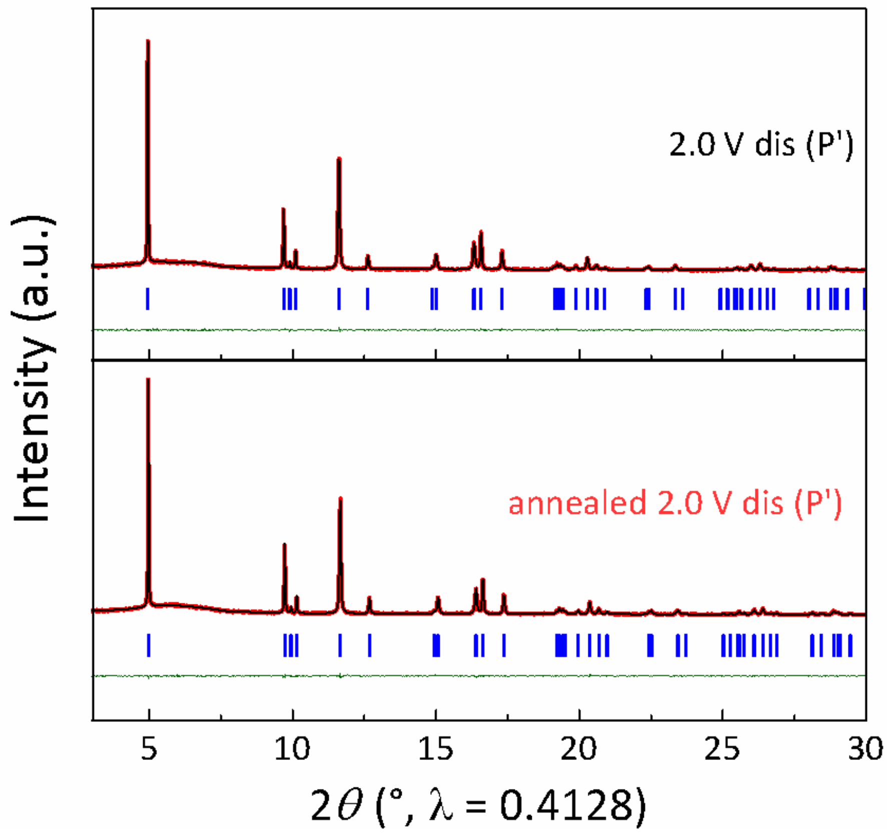

Supplementary Figure 14 Heat-driven structural evolution of Li-rich NMC. Rietveld refinements of the synchrotron XRD patterns of Li-rich NMC collected at 2.0 V discharge state after 4.6 V charge before (denoted as "2.0 V dis (P')") **(a)** and after (denoted as "annealed 2.0 V dis (P')") **(b)** annealing under Ar atmosphere at 250 °C for 1 h. The red line, black line and bottom green line represent the observed, calculated XRD patterns and the difference, respectively. Vertical blue tick bars mark the Bragg positions in the $R\bar{3}m$ space group.

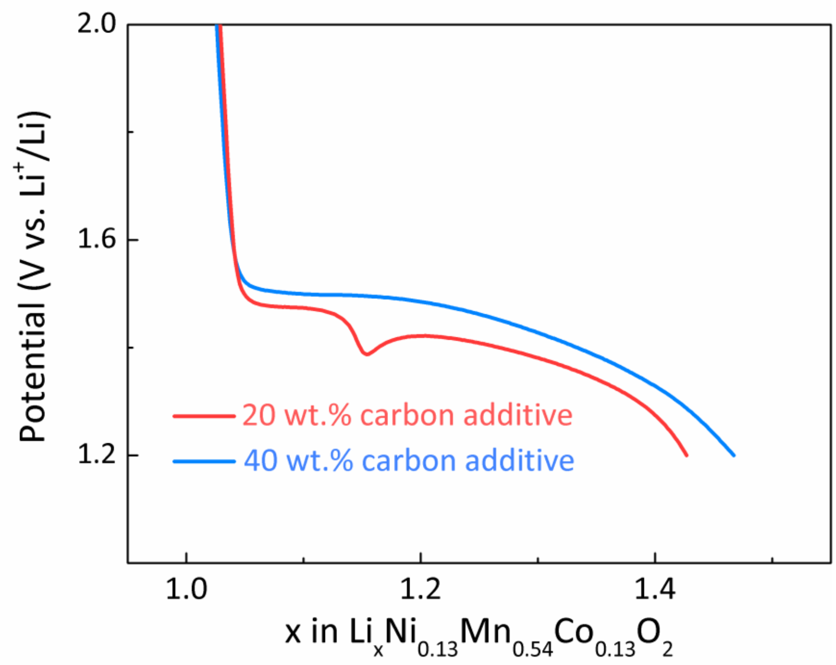

Supplementary Figure 15 The initial discharge curves at low-potential region of Li-rich NMC mixed with 20 wt.% (red line) and 40 wt.% (blue line) carbon super P cycled within a potential window of 4.8 – 1.2 V at a current rate of C/5.

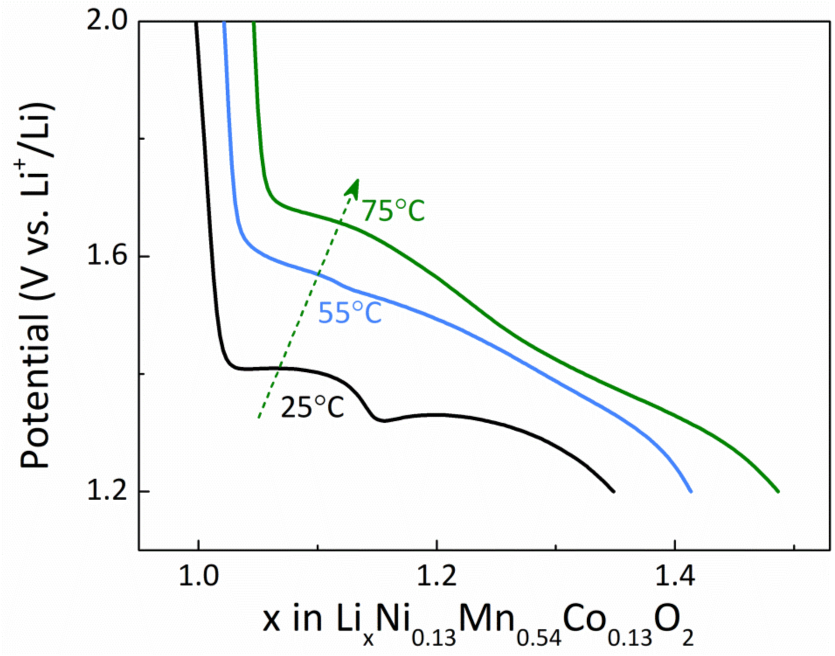

Supplementary Figure 16 The initial discharge curves of Li-rich NMC at low-potential region: three identical cells were charged to 4.8 V, prior to be discharged with a CC protocol at various temperatures of 25 ℃ (black line), 55 ℃ (blue line) and 75 ℃ (green line).

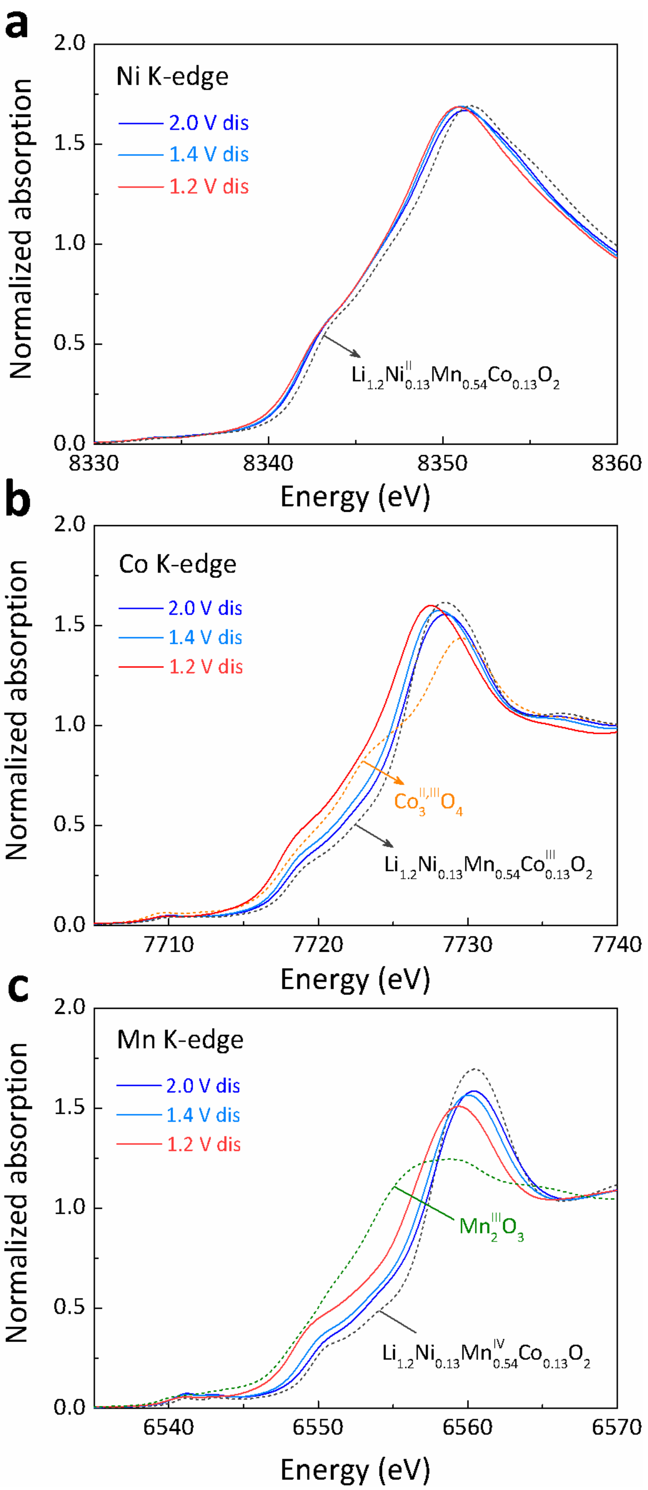

Supplementary Figure 17 Evolution of transition metal oxidation state on deep discharging from 2.0 to 1.2 V. Normalized XANES spectra at the (**a**) Ni K-edge, (**b**) Co K-edge and (**c**) Mn K-edge for Li-rich NMC samples which were identically charged to 4.8 V prior to be discharged to 2.0 V (blue line), 1.4 V (cyan line) and 1.2 V (red line). The spectra for $Li_{1.2}Ni_{0.13}Mn_{0.54}Co_{0.13}O_2$, Co_3O_4, and Mn_2O_3 are given as references.

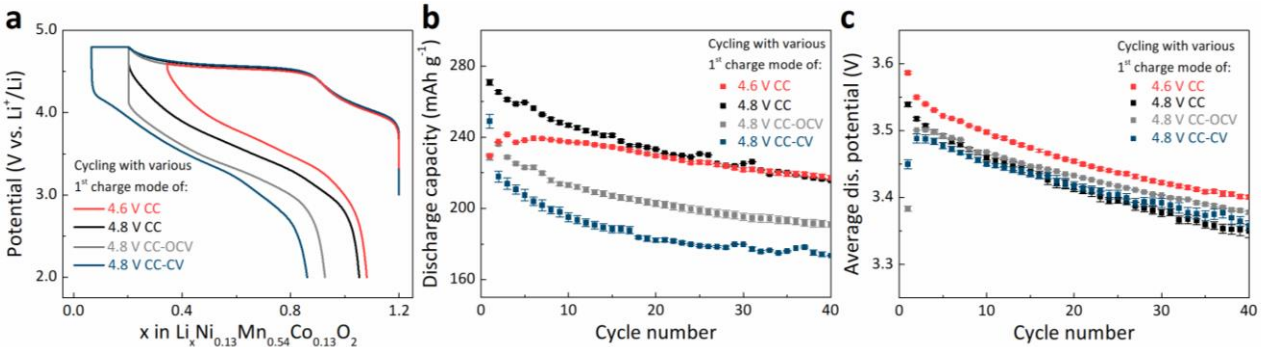

Supplementary Figure 18 Electrochemical performance of Li-rich NMC cycled with various first-charge protocols. a, The first charge-discharge curves of Li-rich NMC cycled at C/3 with different protocols. One cell was cycled to 4.8 V and then directly discharged to 2.0 V, denoted as "4.8 V CC" (black line), whereas an intermediate OCV step for 120 h (grey line, denoted as "4.8 V CC-OCV") or CV hold at 4.8 V for 8 h (blue line, denoted as "4.8 V CC-CV") was respectively imposed to the other two cells. After the first cycle, all the three cells were identically cycled between 4.8 and 2.0 V without neither OCV nor CV step, and their retention profiles of the average discharge potential and the discharge capacity were presented in panel **b** and **c**, respectively. Note that the colors are quoted the same as in panel **a**. The first charge-discharge curve and the retention profiles of the discharge capacity and discharge potential recorded for the cell that was cycled between 4.6 and 2.0 V were also included for the sake of comparison (denoted as "4.6 V CC", red lines).

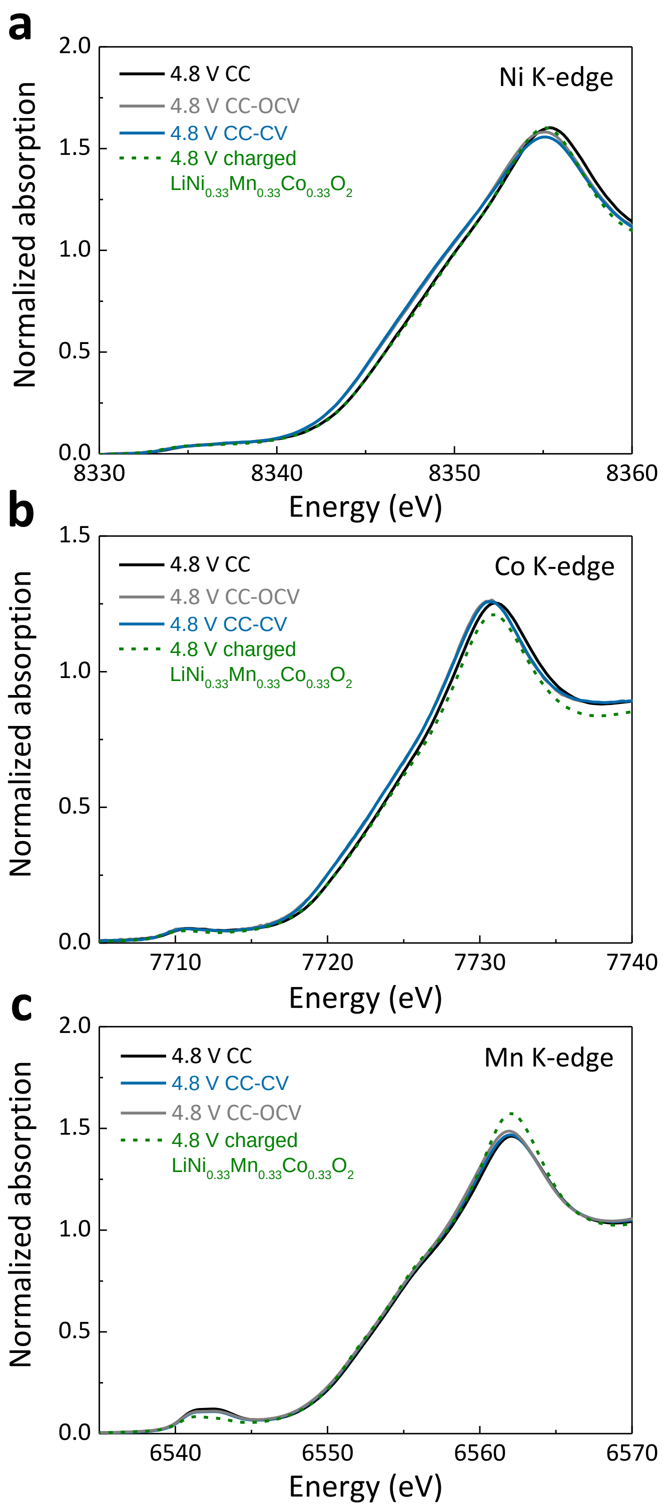

Supplementary Figure 19 Evolution of transition metal oxidation states on deep oxidation. Normalized XANES spectra at the (a) Ni K-edge, (b) Co K-edge and (c) Mn K-edge for Li-rich NMC samples which were charged to 4.8 V (black line), and charged to 4.8 V with an additional OCV step (grey line) or CV hold (blue line) for 5 h. The spectra for $LiNi_{0.33}Mn_{0.33}Co_{0.33}O_2$ collected at 4.8 V charge state are given as references.

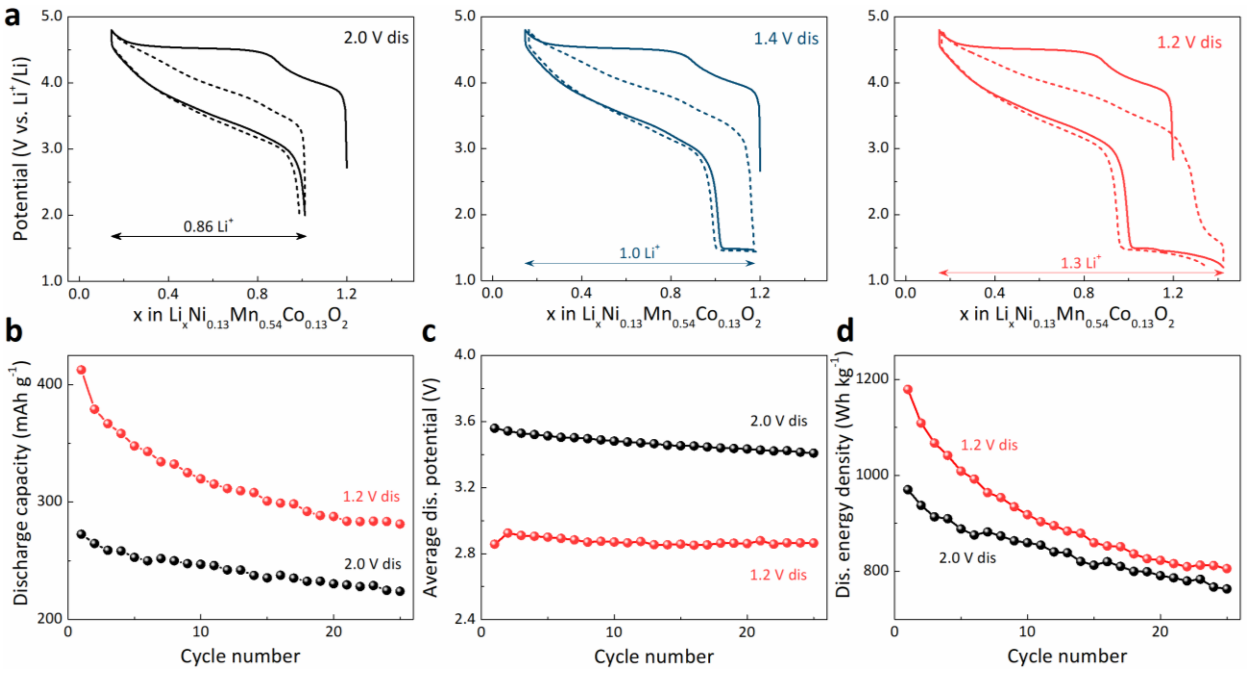

Supplementary Figure 20 **a,** The first (solid lines) and second (dash lines) charge-discharge curves of Li-rich NMC cycled at C/5 by fixing the charge cut-off potential at 4.8 V, while varying the discharge cutoff potential from 2.0 V (black lines), 1.4 V (blue lines) to 1.2 V (red lines). Retention profiles of the discharge capacity (**b**), the average discharge potential (**c**), and the discharge energy density (**d**) of Li-rich NMC upon discharging to 2.0 V (black symbols) and 1.2 V (red symbols).

Supplementary Table 1 Crystallographic data and atomic positions of the pristine Li-rich NMC phase determined from Rietveld refinement of synchrotron XRD pattern.

Pristine Li-rich NMC phase ($O3 – Li_{1.2}Ni_{0.13}Mn_{0.54}Co_{0.13}O_2$)						
Space group: $R\bar{3}m$						
	a (Å)		b (Å)		c (Å)	V (Å^3)
	2.85396(2)		2.85396(2)		14.25381(15)	100.545(2)
χ^2 = 9.09, Bragg R-factor = 5.49 %						
Atom	Site	x/a	y/b	z/c	Occupancy	B_{iso} (Å^2)
Li	3*a*	0	0	0	1	0.54(7)
Ni/Mn/<br>Co/Li	3*b*	0	0	0.5	0.13/0.54/<br>0.13/0.20	0.132(8)
O	6*c*	0	0	0.24105(8)	1	0.351(18)

Supplementary Table 2 Estimation of the O content in charged Li-rich NMC phases. The evolved O_2 and CO_2 amounts are deduced from the same cells presented in Supplementary Fig. 9 based on the ion currents recorded respectively for m/z = 32 and m/z = 44. The total extraction of O atom from the material was estimated by 1) only considering the recorded O_2 amounts, and 2) assuming that CO_2 evolved at high potentials (denoted as high V CO_2) also comes from the released O_2 reacting with electrolyte, as suggested by Strehle *et al.*[1]

	O_2 amount (µmol g^{-1})	CO_2 amount (high V, µmol g^{-1})	x in Li_yTMO_x (considering O_2)	x in Li_yTMO_x (considering O_2 + high V CO_2)
CC	269.5	45.8	1.95	1.95
CC-OCV	383.4	40.7	1.93	1.93
CC-CV	751.0	126.0	1.87	1.85

Supplementary Table 3 Crystallographic data and atomic positions of the phase A' determined from combined Rietveld refinements of synchrotron XRD and neutron powder diffraction (NPD) patterns.

**CC-8 h CV charged Li-rich NMC phase (Phase A': O3 – $Li_{0.05}Ni_{0.14}Mn_{0.58}Co_{0.14}O_2$)**						
Space group: $R\bar{3}m$						
	a (Å)		b (Å)	c (Å)	V (Å^3)	
SXRD	2.84194(11)		2.84194(11)	13.9722(14)	97.730(11)	
NPD	2.8297(6)		2.8297(6)	13.878(7)	96.24(6)	
χ^2 = 5.1, Bragg R-factor = 14.5 % (SXRD), Bragg R-factor = 21.3 % (NPD)						
Atom	Site	x/a	y/b	z/c	Occupancy	B_{iso} (Å^2)
Mn	3a	0	0	0	0.083(2)	0.38(3)
Ni/Mn/ Co/Li	3b	0	0	0.5	0.14/0.499(2)/ 0.14/0.056	0.38(3)
O	6c	0	0	0.2422(2)	1	1.75(4)

Supplementary Table 4 Crystallographic data and atomic positions of Li-rich NMC phase charged to 4.8 V followed by 120 hours OCV determined from Rietveld refinement of synchrotron XRD pattern.

**4.8 V charged Li-rich NMC phase after 120 h resting ($O3 – Li_{0.2}Ni_{0.13}Mn_{0.56}Co_{0.13}O_2$)**						
Space group: $R\bar{3}m$						
	a (Å)		b (Å)		c (Å)	V (Å^3)
	2.84525(3)		2.84525(3)		14.2057(4)	99.595(3)
χ^2 = 1.76, Bragg R-factor = 10.1 %						
Atom	Site	x/a	y/b	z/c	Occupancy	B_{iso} (Å^2)
Li	3a	0	0	0	0.2	0.449(8)
Mn	3a	0	0	0	0.081(2)	0.449(8)
Ni/Mn/ Co/Li	3b	0	0	0.5	0.13/0.46/ 0.13/0	0.449(8)
O	6c	0	0	0.24034(10)	1	2.11(3)

Supplementary References

1. Strehle, B. *et al.* The Role of Oxygen Release from Li- and Mn-Rich Layered Oxides during the First Cycles Investigated by *On-Line* Electrochemical Mass Spectrometry. *J. Electrochem. Soc.* **164**, A400−A406 (2017).
